# *In Vivo* Colocalisation of *oskar* mRNA and *Trans*-Acting Proteins Revealed by Quantitative Imaging of the *Drosophila* Oocyte

**DOI:** 10.1371/journal.pone.0006241

**Published:** 2009-07-14

**Authors:** Musa M. Mhlanga, Diana P. Bratu, Auguste Genovesio, Agata Rybarska, Nicolas Chenouard, Ulf Nehrbass, Jean-Christophe Olivo-Marin

**Affiliations:** 1 Unité de Biologie Cellulaire du Noyau, Département de Biologie Cellulaire et Infection, Institut Pasteur, Paris, France; 2 Department of Biological Sciences, Hunter College, City University of New York, New York, New York, United States of America; 3 Unité d'Analyse d'Images Quantitative, Département de Biologie Cellulaire et Infection, Institut Pasteur, Paris, France; Oregon State University, United States of America

## Abstract

Efficient mRNA transport in eukaryotes requires highly orchestrated relationships between nuclear and cytoplasmic proteins. For *oskar* mRNA, the *Drosophila* posterior determinant, these spatio-temporal requirements remain opaque during its multi-step transport process. By *in vivo* covisualization of *oskar* mRNA with Staufen, its putative trafficking protein, we find *oskar* mRNA to be present in particles distinct from Staufen for part of its transport. *oskar* mRNA stably associated with Staufen near the posterior pole. We observe *oskar* mRNA to oligomerize as hundreds of copies forming large particles which are necessary for its long range transport and localization. We show the formation of these particles occurs in the nurse cell nucleus in an Hrp48-dependent manner. We present a more refined model of *oskar* mRNA transport in the Drosophila oocyte.

## Introduction

The transport and localization of mRNAs to particular sites within cells is of broad biological importance and is essential in diverse processes such as growth and differentiation, asymmetric cell division, long-term memory formation, axon guidance and the establishment of the basic body axes [1], [2]. Coupled with spatially regulated translation of mRNA, these processes are an important mechanism of post-transcriptional regulation of gene expression throughout eukaryotic life [3]. Messenger RNA is trafficked from sites of transcription in the nucleus to specific destinations in the cytoplasm via a heterogeneous nuclear ribonucleoproteins, (hnRNPs) or mRNA-protein complexes (mRNPs). The *Drosophila melanogaster* oocyte offers an ideal model system to study mRNP interactions with proteins involved in transport [2]. Though genetic data has identified hnRNPs and other cytoplasmic proteins to be key during the transport process, for many their specific roles remain unknown. In the oocyte, the composition of the mRNP particle during transcription, transport, and eventual translation, ultimately determines the successful formation of the anterior-posterior and dorso-ventral body axes [4], [5]. In the case of *oskar* mRNA (*osk*), which specifies the inchoate pole plasm as well as abdominal and germline determinants [6], [7], its transport, spatial positioning and eventual translation require the occurrence of precise nuclear and cytoplasmic events [8], [9], [10], [11]. Several proteins that repress translation of *osk* during its transport have also been identified. Many act to aggregate *oskar* transcripts during transport, a process that is likely conserved for many other mRNAs across species [10], [12], [13], [14], [15]. Indeed, more trans-acting factors are known for *osk* than for any other localized transcript [16].

A protein central to *osk* transport, and the first identified affecting its posterior localization is Staufen [17]. It has also been shown that Staufen is important in the transport of mRNAs in numerous cell types in *Drosophila* and mammals, the best studied of these being the *Drosophila* oocyte [18]. Thus understanding the role of Staufen is of broad importance, as its role in mRNA transport is apparently conserved in differing cell types across different species [19]. Staufen is postulated to interact directly with *osk* either during or just after the transcript exits from the nurse cell nucleus, and believed to escort *osk* to the posterior pole of the oocyte [17], [20], [21]. A contemporaneous study by Zimyanin et al. has shown Staufen to interact with *osk in vivo* though the precise spatial location within the oocyte of their interaction during the localization process remains unknown [22]. The localization of *osk* is impaired in Staufen mutants [17]. Though Staufen has not been found to bind specifically to *osk in vitro*, two lines of evidence are commonly used to suggest that it does so *in vivo*: mutations in Staufen abrogate the localization of *osk* [20], [21], and secondly, Staufen is colocalized with *osk* at the oocyte posterior implying a functional link between the two [23]. Staufen is also required for the translation of *osk* to Oskar protein [14], [20], [24].

How Staufen and other proteins contribute to *osk* transport is poorly understood. Understanding the spatio-temporal relationship between *osk* and these *trans*-acting proteins necessitates approaches permitting the direct observation of such dynamic events *in vivo* [16], [25]. Advances that facilitate the tracking and covisualization of *trans*-acting factors with individual mRNP particles in real time, such as those introduced by Zimyanin et al, permit the precise description of transport events and how they are influenced by these factors [22]. They assist in deciphering the highly orchestrated behavior between mRNA and proteins addressing several questions, including: (i) the biophysical properties of the transport from the point of transcription to eventual localization (ii) the temporal requirement of specific nuclear or cytoplasmic proteins, and (iii) the principal nature and composition of the *osk* particle during its transport.

Here we begin to answer these questions by relating for the first time the spatial position where Staufen begins to escort *osk* during localization in real-time. We covisualize *native* individual *osk* particles and Staufen *in vivo*. We show *osk* does not require Staufen to reach the posterior region of the oocyte, strongly suggesting Staufen is necessary for only a terminal step in the localization of *osk*. We demonstrate and directly observe for the first time what has only been hypothesized, that *osk* is transported and anchored in large, higher order RNP assemblies that are constantly reshaped in their form during transport. We find that the nurse cell nuclear protein Hrp48 plays a key role in the formation of these structures. We confirm recent studies that *osk* transport in the nucleus can be modeled by what we term diffusive transport whilst in the nurse cell and the oocyte it occurs via super-diffusive transport. Thus we build upon contemporaneous studies to develop a more refined model of long-range transport of *osk*.

## Results

### Dynamics of *oskar* mRNA in oocytes

We visualized endogenous *oskar* mRNA (*osk*) using molecular beacons. Molecular beacons are internally quenched hairpin shaped oligonucleotides probes that become fluorescent upon hybridization with their target sequence [26]. In a previous report, we showed that these probes can specifically light up *osk* while ignoring other contents of the oocyte [27]. In that study, several controls demonstrated the extreme specificity of these probes: when *osk* localization is disrupted, the posterior signal is lost; when the 3′ UTR of *bicoid* mRNA (a mRNA localized to the anterior via its 3′ UTR) is transplanted on the *osk* coding sequence, the signal emanates from the anterior of the oocyte; and when two molecular beacons designed to bind side-by-side on *oskar* mRNA as a FRET (Fluorescence Resonance Energy Transfer) pair are introduced in oocytes, a clear posterior localization is observed, indicating specific hybridization and detection of *osk* with localization unperturbed. Furthermore, the target specificity of molecular beacons has been confirmed in various other cellular contexts [28], [29].

**Figure 1:**
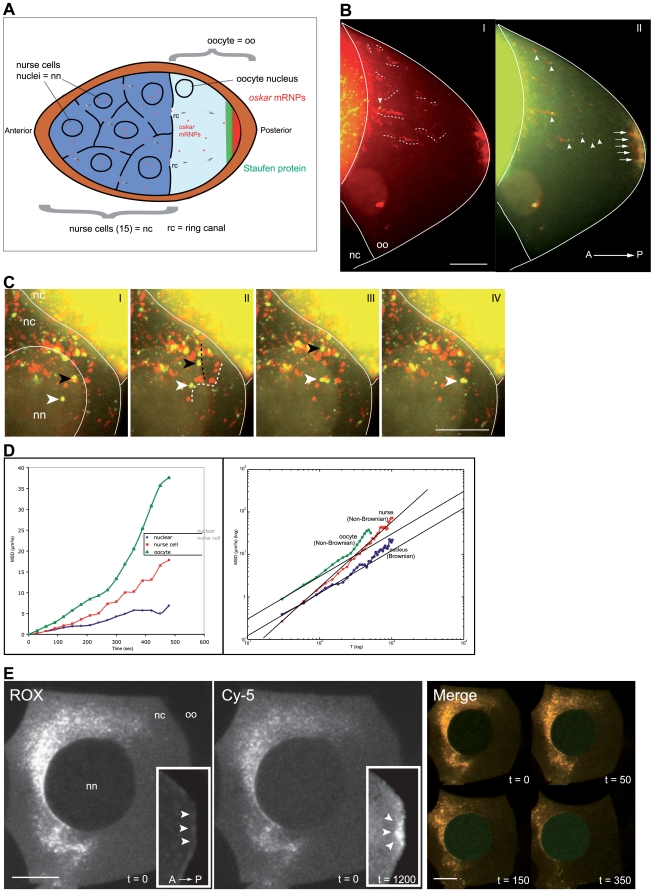
*Oskar* mRNP dynamics during stage7-10 oocytes. (A) Model of *oskar* mRNA transport in stage 7 to 10 oocyte. *Oskar* mRNA is synthesized in the nurse cell nuclei, transported through the ring canals interconnecting the nurse cells and the oocyte, and ultimately localized to the posterior of the oocyte in a tight crescent. *Oskar* mRNA (red) colocalizes with Staufen protein (green) at the oocyte's posterior. (B) A cocktail of molecular beacons labeled with Cy5 fluorophore and complementary to several regions of native *oskar* mRNA was injected into the nurse cell nuclei of a stage 9 egg chamber. The transport of *oskar* mRNA from the nurse cell nucleus to the posterior cortex of the oocyte is directly visualized *in vivo*. We acquired images continuously taking 18 optical slices of 1.2 µm in size over a period of 20 min. A central optical slice projected over 200 time points is represented in red. The tracks of several particles are traced in the oocyte in white dash lines. The first and last time point frames (green&yellow) (panel I and II, respectively) are merged with the time projection image to indicate the position of the particles at the beginning and the end of the tracked paths inside the oocyte. These tracks reflect directional movement towards the posterior pole. Individual particles are observed transiting through the ring canal, accumulating over time in the oocyte, and forming a crescent of *oskar* mRNA associated with the posterior (arrows). See Movies S2 & S4. (C) Observation of *oskar* mRNA export from the nucleus into the nurse cell cytoplasm over 10 min. Images of independent time points (I–VI) (green) are merged with a time-projected image (red). The tracks of two particles are traced over time (black and white arrowheads). See Movies S1 & S3. (D) Quantitative analysis of *oskar* mRNP dynamics in three compartments of the *Drosophila* egg chamber by automated multiple particle tracking over 30 minutes. Within the nucleus we calculate the coefficient of diffusion to be 1.09 (energy independent-subdiffusion). In the nurse cell cytoplasm we find this to be 1.3 (over-diffusive). In the oocyte chamber we observe this to be energy dependent. (E) The co-injection of *in vitro* transcribed ROX-labeled *oskar* RNA with an *oskar*-specific molecular beacon labeled with Cy5 into a wild type nurse cell. The nurse cell nuclei (nn) and oocyte (oo) were imaged every 10 sec over 20 min. Upon co-injection, a population of probes hybridized to the transcribed RNA revealing particles almost instantaneously (Cy5 panel, t = 0). Free, unbound probes diffuse and become sequestered within the nucleus in minutes (green in Merge panel). In the oocyte, they bound tightly to localized endogenous *osk* at the posterior pole (Cy-5 panel inset, t = 1200). The *oskar*-ROX signal was distributed throughout the nurse cell cytoplasm, never in the nucleus, and with very little accumulation in the oocyte and at the posterior end (ROX panel inset, t = 1200). Posterior is oriented to the right. Scale bar is 25 µm in (B) and 10 µm in (C, E). N>100.

We used a cocktail of four molecular beacons in which each molecular beacon could bind to a different region in the *osk* coding sequence. Using carefully controlled concentrations resulted in higher fluorescence intensity and lower background signals, thus permitting high speed imaging in 3-D (Methods, Supporting Information). We used rapid Nipkow disk laser confocal microscopy with piezo control over 3D optical slicing. In our first set of experiments, we microinjected the molecular beacon cocktail into the cytoplasm of a nurse cell proximal to the oocyte (see Figure 1A for the organization of egg chamber). The *in vivo* dynamic behavior of *osk* in stage 7 to 10 oocytes was imaged over the entire oocyte. The dynamics of *osk* are shown in Figure 1B and C, and Movies S1, S2, and S3. *Osk* was visible as discrete “particles” of different shapes and sizes. We tracked hundreds of particles with very little loss in fluorescence intensity over the period of the assay, capturing the dynamics of *osk* from its nuclear export to its localization at the posterior of the oocyte (Figure 1). An analysis of the intensity of these particles indicated that each particle contained a large number of mRNA molecules (Supplementary Information). To be able to accurately characterize and quantify the behavior of what appeared to be several classes of *osk* transcripts, we used an advanced mathematical algorithm [30], [31], [32], [33], [34] integrated into an image analysis software (QUIA) developed specifically for the purpose of the detection and tracking of such particles *in vivo*.

Single particle analysis was used to characterize the mobility of mRNP particles in different compartments. The relationship between mean square displacement (MSD) and time interval can be used to classify the motion of a particle as ‘classical diffusion,’ ‘confined diffusion’ or ‘active transport’ [35]. Most studies analyze particle motion in terms of diffusion coefficients, which implicitly assumes that the motion is Brownian. When we subjected our data to such analysis, we were only able to fit the nuclear component of mRNA motion to Brownian motion diffusion analysis (see Supporting Information). The motion of *oskar* mRNA in the nurse cell and especially the oocyte compartments were non-Brownian in nature. Our quantitative characterization of *osk* dynamics in these two compartments showed it to be super-diffusive (see Supporting Information) [36], [37]. The results of our analysis of a large number of particles in different regions of the egg chamber are presented in Figure 1D.

The exponent alpha, extracted from the power-law fitting the MSD curves, reflects the motion dynamics (α = 1 corresponds to Brownian motion). Values of α greater than 1 (termed “super-diffusion”) are indicative of directed motion and those less than 1 relate a motion that is freely diffusive and undirected [36], [37]. In the nurse cell cytoplasm, *osk* motion had an α = 1.4465, in oocytes α = 1.166, and in the nucleus we determined the diffusion coefficient of *osk* to be 0.0124 µm^2^/s. Modeling of MSD to these specific types of non-Brownian motion is further described in the Supporting Information section. The behavior of *osk* in the nucleus was similar to the mobility of other mRNP complexes in the nucleus of cultured cells [29], [38]. In contrast, the motion of *osk* in the oocyte and nurse cell cytoplasm is attributable to active transport, which could either occur via molecular motors or movement under the influence of cytoplasmic flows.

As part of this study, we also performed a number of experiments to understand the kinetics of diffusion of molecular beacon probes within the egg chamber and the rates of hybridization to *oskar* mRNA (Figure 1E & Supporting Information: Figure S1). We co-injected *in vitro* transcribed ROX-labeled *oskar* RNA with an *oskar*-specific molecular beacon labeled with Cy5 into a wild type nurse cell. We determined whether the fluorescent signals we observed localized at the posterior of the oocyte were due to transiting *osk* particles, rapidly diffusing molecular beacons, or a combination of both. A population of probes hybridized to the transcribed RNA whilst unbound probes were free to diffuse, with some being taken up into the nucleus within minutes. In the oocyte, the probes bound tightly to localized endogenous *osk* at the posterior pole. *oskar*-ROX signal was distributed throughout the nurse cell cytoplasm, never in the nucleus and scant accumulation in the oocyte. Since *in vitro* transcribed *oskar*-ROX does not undergo nuclear processing, it failed to reach the posterior [9]. These studies are further described and documented in the Supporting Information section, bringing clarification about the advantages of using molecular beacons over other fluorescently labeled probes.

We also observed, as has been noted in numerous studies [28], [29], [39], that the nurse and oocyte nuclei had a tendency to take up molecular beacons. But despite this occurrence of nuclear uptake, which has also been observed by other investigators with small DNA molecules [40], [41], [42] the nuclear fluorescent signals decrease over the length of the assay. Therefore in the nucleus, molecular beacons are able to bind to their mRNA targets, and are exported together [29].

### Covisualization of *oskar* transcript with Staufen protein during transport *in vivo*

A high degree of interplay between nuclear and cytoplasmic proteins is apparent in the correct transport and localization of *osk* [2], [25], [43]. *osk* transport implicates three types of *trans*-acting factors whose link to trafficking mechanisms are unknown: (1) factors loaded co-transcriptionally (i.e. hnRNPA/B family member Hrp48 and Bruno), and during splicing of the first intron, such as the exon junction complex members (EJC) (i.e. Y14 and Mago nashi), (2) factors loaded in the cytoplasm which may connect *osk* to active transport pathways (i.e. Staufen), and finally (3) those factors loaded when *osk* reaches the posterior compartment to spatially control translation (as yet unidentified).

Some models propose that Staufen escorts *osk* to the posterior tip of the oocyte [17]. These models result from studies in which mutations in Staufen lead to mislocalization of *osk* to the anterior end, the middle of the oocyte and with slight posterior localization [20], [44]. Indeed contemporaneous studies have measured the mobility of GFP-labeled Staufen and found it to be slightly discordant with that of osk particles, though the regions of the oocyte over which these measurements are taken are unclear [22]. Efforts to demonstrate biochemically the direct binding of Staufen to *oskar* mRNA are notably absent in the literature. Therefore, in order to investigate the role of Staufen protein in the transport of *osk*, we co-visualized the two molecules in live oocytes and studied their mobility during oocyte development. To visualize Staufen protein in live oocytes, we utilized flies in which the natural Staufen gene was replaced with a Staufen-GFP gene (no functional endogenous Staufen, identical to the transgenic line used by [45]). This addition to Staufen has been shown to preserve the functionality of the protein retaining normal localization and leaving the posterior localization of *osk* unaltered.

**Figure 2:**
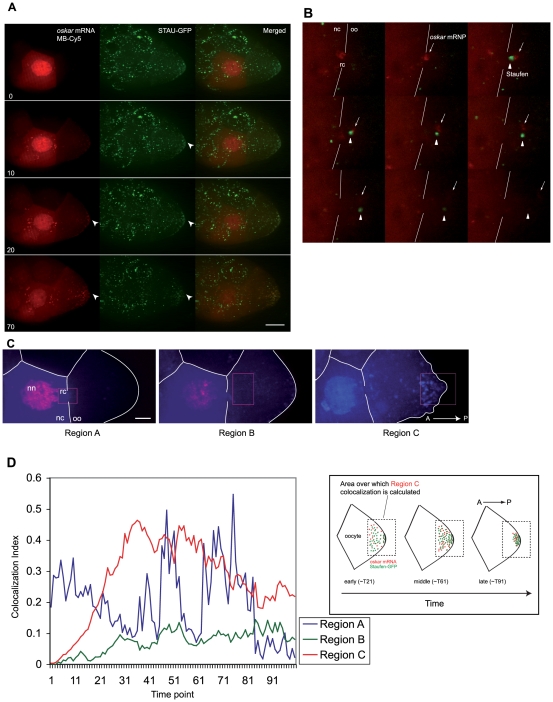
Covisualization of *oskar* mRNP with *trans*-acting protein Staufen *in vivo*. (A) Direct *in vivo* visualization of the dynamics of *oskar* mRNA (red) labeled with Cy5 molecular beacons, and Staufen protein (green) labeled with GFP over 90 minutes. Each time point panel (min) is merged, indicating the enrichment and colocalization of the two signals at the oocyte's posterior (arrowhead). Both channels were acquired with settings as described within the text. These are z-projections that have been adjusted to reduce background contributions. See Movie S5. (B) Optical slice of the transit of *oskar* mRNP and Staufen protein from nurse cell chamber to the oocyte through the ring canal. Across the panels (each 3 seconds apart), *oskar* mRNP (red) and Staufen protein (green) persist as distinct separate particles as they exit the ring canal, after being in very close proximity at the ring canal. See Movie S8. (C) Three defined regions of the oocyte in (A), which were used for colocalization estimation analysis. Region A includes the nurse cell nucleus, the ring canal and a small anterior portion of the oocyte. Region B covers the anterior and central region of the oocyte. Region C includes the posterior tip and posterior region of the oocyte. The color representation is of a ‘heat map’ of *osk*. (D) Graphical representation of the colocalization index for predefined regions A, B and C. Region A shows large fluctuations in colocalization as *oskar* mRNA and Staufen protein undergo frequent contacts as *oskar* mRNA is exported from the nucleus and encounters large concentrations of Staufen protein in the cytoplasm. The persistently low colocalization index in region B is indicative of little or no colocalization. For region C, it rises rapidly from time point 11 to 31 reflecting colocalization, and decreases over time as the particles associate tightly to the posterior pole (cartoon on the right). Posterior is oriented to the right. Scale bar is 25 µm in (A,C) and 10 µm in (B). N>40.

We co-visualized the two molecules in stage 7 to 10 oocytes (Figure 2 & Movie S5). *Drosophila* oocytes expressing the Staufen-GFP transgene, display Staufen protein particles throughout the oocyte and nurse cells, in a pattern similar to what has been previously reported using antibodies against Staufen and GFP-fusion proteins [45], [46]. Given the near ubiquitous localization pattern of Staufen, we expected the interaction with *osk* to occur immediately upon exit of *osk* from the nucleus. Contrary to our expectations, *osk* and Staufen persisted as separate particles throughout the transport process until they reached an accumulation point near the mid-posterior of the oocyte chamber (Figure 2A, Movie S5). Though *osk* and Staufen protein particles underwent numerous collisions and physical encounters prior to this point, no stable complex between the two was observed (Figure 2A, B). This suggests that *osk*'s association with Staufen protein, as observed by simple colocalization studies, is able to occur only within the oocyte chamber, and at a late stage in the transport process. A dissimilar phenotype is observed when Barentsz-GFP is co-visualized with *osk* (Supporting Information: Figure S3). These data suggest that Staufen does not play a role in the early trafficking steps of *osk* transport, but rather in the terminal stages of transport.

To correctly position each detected *osk* particle in time and space relative to each detected particle of Staufen over the volume of the entire oocyte, we analyzed our results using an advanced mathematical algorithm, termed ‘colocalization estimation’ (technical description provided in Supplementary Data S1). This analysis allowed us to dynamically quantify the degree of colocalization between *osk* and Staufen. We quantified the intensity of fluorescence emitted in two wavelengths simultaneously, the first from molecular beacons bound to *osk* and the second from Staufen-GFP (Figure 2A).

The colocalization estimation of the *osk* and Staufen protein was performed in three regions (Figure 2C). Region A was centered on a ring canal most proximal to the oocyte chamber and included portions of the oocyte and nurse cell chambers. Region B was in the anterior of the oocyte chamber just beyond the ring canal. Region C was the posterior extremity of the oocyte centered upon the tip of the oocyte where *osk* and Staufen protein both colocalized (arrowheads in Figure 2A, Movie S3). For each of these regions, we determined a colocalization index (Figure 2D) as a function of time. In region A, we observed the colocalization index to fluctuate greatly, with fleeting collisions of *osk* and Staufen-GFP trafficking through the ring canal marking the peaks of the colocalization. This suggested that though *osk* and Staufen protein engage in frequent collisions prior to and just after traversing the ring canal, these interactions do not result in their stable association. Region B displayed the lowest amount of colocalization. Thus, as *osk* passed from the nurse cell into the anterior portion of the oocyte, colocalization with Staufen did not increase strongly suggesting that association with Staufen does not occur in this region. Further, it disfavors a model where *osk* is ‘fetched’ from the anterior and transported to the posterior of the oocyte due to an interaction with Staufen, as has been posited by some [46], [47]. In region C, the results were markedly different, as the amount of *osk* accumulated in the center of the oocyte; the index of colocalization with Staufen protein rose remarkably and maintained a high level (Figure 2A, D). *Osk* and Staufen protein had the greatest colocalization estimation when they were in the posterior half of the oocyte. These data suggest Staufen protein is not directly in association with *osk* during the trafficking process, reinforcing our earlier assertion that it is most likely involved in the posterior localization phase.

### *Osk* dynamics in Staufen mutant oocytes

**Figure 3:**
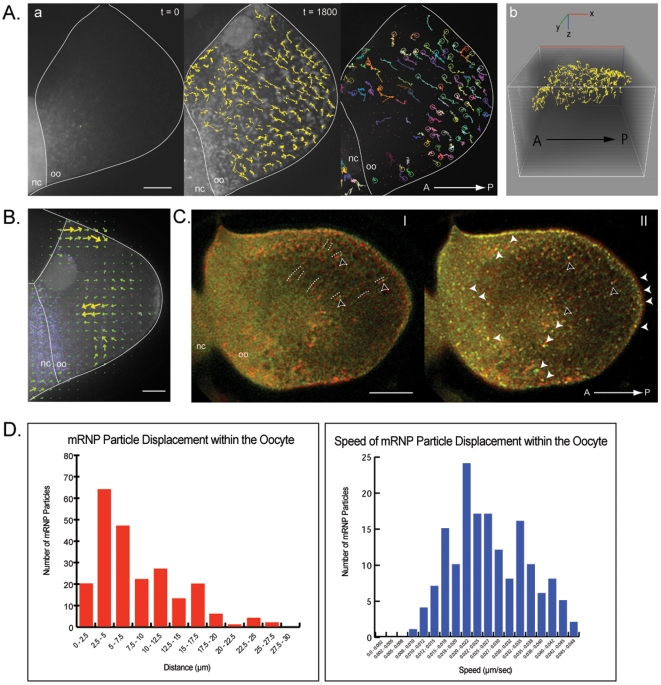
*Oskar* mRNA dynamics in Staufen mutant oocytes. Molecular beacon cocktail was microinjected in a nurse cell (A) or an oocyte (C) of egg chambers mutant for Staufen protein. *oskar* mRNA was imaged over approximately 50 minutes. (A) *oskar* mRNA particles were tracked in two (a) and three (b) dimensions. The end point of each particle track highlighted in the 2D colored panel is represented by circle. (B) High resolution 3D tracking of wild-type oocyte where velocity and trend of flow of *osk* particles is overlaid on the oocyte by arrows, as compared to Stau mutant. Large arrows represent faster velocities and stronger flows, whilst smaller arrows represent slower velocities. *Osk* particles seem to have a posterior bias, which concentrates at the posterior tip with both flows then seeming to “rebound” from the posterior to the center creating a strong central anterior flow. Since *osk* in wild-type is captured at the posterior, this effect is minimized. In Stau mutants, where *osk* is not captured at the posterior, this anterior flow dominates and *osk* eventually acquires an anterior localization. (C) The first and last time point frames (green&yellow) (panel I and II, respectively) are merged with the time projection image (red) to indicate the position of the particles at the beginning and the end of the tracked paths inside the oocyte. Tracks of several particles are traced by white dotted line. The black arrowheads indicated the beginning (in panel I) and end (in panel II) points of those particles defining the tracks. *Oskar* mRNA is distributed throughout the oocyte including at the posterior and anterior of the oocyte (white arrowheads). No crescent-like localization is observed as in wild type oocytes (B). (D) The tracking data shows the particles over the time course as they appear to retreat toward the anterior once they reach the posterior as if they are no longer anchored there. See Movies S6. Posterior is oriented to the right. Scale bar is 25 µm. N = 23.

When stage 7–10 oocytes mutant for Staufen are probed *in situ* with respect to *osk*, it is observed at high concentration at the anterior [20], [44]. This suggests at least two possibilities, either in a Staufen null mutant there are pleiotropic effects in the oocyte that cause *osk* to accumulate at the anterior, or that the bulk of *osk* requires Staufen protein to proceed beyond the anterior of the oocyte. Our previous observation implied that *osk* should be able to proceed to at least the center of the oocyte and perhaps beyond before requiring association with Staufen protein. To address this question we imaged, with high resolution, the dynamics of native *osk* in stage 7–10 Staufen mutant oocytes. We specifically were interested in observing the specific point at which *osk* transport is arrested and fails to accumulate at the posterior of the oocyte. We directly imaged the transport of *osk* through the ring canal and into the oocyte from the nurse cell (Figure 3A, Movie S6). We observed that *osk* continued its journey and became distributed throughout the oocyte chamber. It persisted in large particles but never became localized in its characteristic large crescent at the posterior tip of the oocyte (compare Figure 3A with Figure 3B). Further, we noted that in the oocyte chamber a greater concentration of *osk* became localized to the anterior than to the posterior in all instances that we examined (n = 23). Further the biophysical characteristics of *osk* motion in the Stau (Figure 3C)) retained its posterior bias, which failed to the “captured” at the posterior.

We observed that during the transport of *osk* transcripts they continued to form the large particles that were similar to those observed in wild-type oocytes. We also observed that when we calculated the absolute displacement of *osk* particles, we found their displacement to be extensive, and the particles were able to occupy large areas of the oocyte chamber including the posterior of the oocyte (Figure 3C, Movie S6). Interestingly when these particles reached the posterior, few remained there and the majority appeared to drift back towards the anterior region of the oocyte, as if they had failed to be “captured” there. Cytoplasmic flows were observable in Staufen mutants and did not appear to differ significantly from wild-type oocytes (Figure 3D). Several groups have made this observation [47], [48], [49]. *Osk* localization and subsequent accumulation at the posterior tip of the oocyte has been shown to require Oskar protein as an anchor and the presence of functional Staufen [20], [50]. Our observations suggest that lack of posterior localization of *osk* is due to disruption in its ability to anchor there rather than its ability to reach the posterior pole. It is also consistent with the failure to produce Oskar protein at the posterior tip of the oocyte as has been observed in Staufen mutants. These data suggest an independence of functional Staufen for trafficking *osk* prior to the center of the oocyte and rather strongly implicates it in a later step in localization where *osk* is translated, facilitating its accumulation at the posterior. It is also consistent with our observation that Staufen and *osk* are only able to interact in the posterior region of the oocyte.

### Extensive reshaping of *oskar* mRNP during transport through ring canal

**Figure 4:**
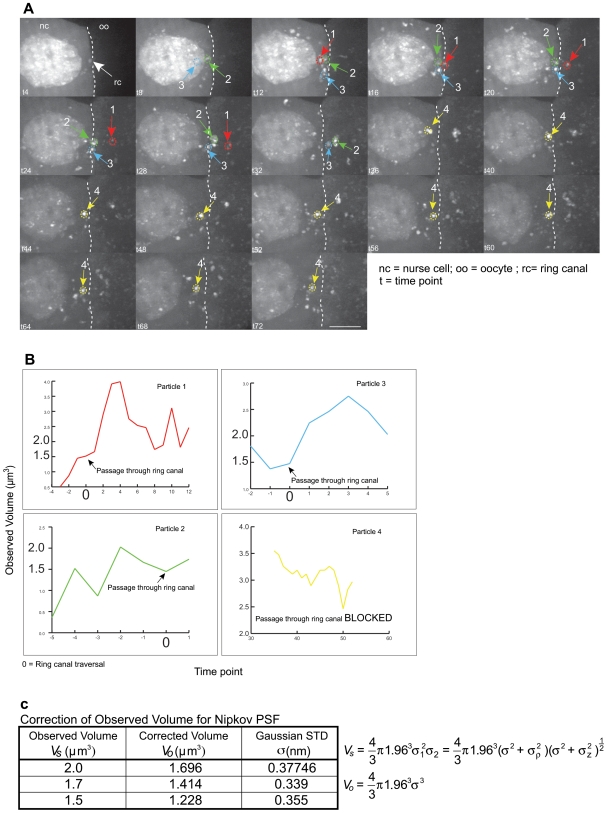
*Oskar* mRNA dynamics through the ring canal are indicative of extensive mRNP remodeling. (A) An example of 4 particles shown undergoing extensive changes in size as they approach and traverse the ring canal. (B) The particles undergo fluctuations in size (represented graphically) until they fall within the permissive volume to allow traversal. These are observed maximum volumes (*V_s_*). (C) These volumes (*V_s_*) are then corrected using the Gaussian function to calculate the standard deviation for the Point Spread Function of the Nipkow spinning disk confocal microscope used. The resulting calculated volumes (*V_o_*) are indicated in the table, with each factor further described in the supporting methods. See Movies S7 & S8. Scale bar is 10 µm.

During our real time observations of the trafficking of *osk*, we found that the shapes of the particles were undergoing alterations in size and shape. Therefore we set out to analyze those changes systematically. We theorized that if indeed *osk* and Staufen protein were interacting prior to the oocyte center, we should observe interactions in the nurse cell compartment. Staufen is distributed widely in the nurse cell compartment. This means it is able to bind *osk* prior to crossing into the ring canal. Such an observed outcome would favor current models of how *osk* and Staufen co-assemble and are transported. Should this be the case, we should observe the transport of this complex containing *osk* and Staufen transiting through the ring canal. We proceeded to image the transport of *osk* and Staufen-GFP through the ring canal. We observed interesting behavior from *osk* particles as they approached the ring canal (Figure 4). *osk* particles appeared to stall in their progression towards the ring canal prior to undergoing a reshaping process. They would alter in size and shape, then re-attempt to cross the ring canal or completely fail to cross the ring canal until undergoing further reshaping (Movies S7 & S8). Throughout the egg chamber the *osk* particle underwent several changes in shape and size during its transport, and persisted as a large oligomer containing, what we have theoretically estimated to be, hundreds of transcripts (Supporting Information). Around the ring canal, several *osk* particles underwent frequent collisions with Staufen particles, though they did not result in permanent associations. Those *osk* particles that seemed unable to cross the ring canal despite remaining in its vicinity for an extended period of time (up to 45 min) did so while remaining mobile. The particles repositioned themselves frequently prior to each attempt at crossing the ring canal (Movie S7). A third class of *osk* particles, shrunk in size or altered in shape as previously described, and then crossed the ring canal as if they had been reshaped to an appropriate size or shape to cross the ring canal (Figure 4, Movie S8 and Supporting Information: Figure S2). In every instance when we observed *osk* together with Staufen-GFP (n>30 flies) we did not find a single example when *osk* persisted as a cocmplex with Staufen-GFP, while entering and exiting the ring canal (Movie S8).

We then analyzed this data with QUIA to understand changes in the estimated volume of each detected *osk* particle (Figure 4B). We were able to determine that every particle of *osk* that crossed from the nurse cell, through the ring canal and into the oocyte, could not exceed a volume of 1.5 to 1.7 µm^3^. Those *osk* particles that appeared to hover in front of the ring canal did so until they reduced some of their volume to reach this size limit (Movie S5). Upon crossing the ring canal, some of these particles could increase in size soon afterwards in the oocyte compartment. The *osk* particles that were stalled on one side of the ring canal unable to cross into the oocyte were all above the 1.7 µm^3^ threshold, and thus remained unable to pass through the ring canal until they underwent the reshaping process. These data strengthened our earlier observations of Staufen and *osk* interactions. They suggest that Staufen protein or *oskar* transcript are incompetent to stably associate with each other prior to reaching a defined region of the oocyte chamber. Our results are, to our knowledge, the first visual confirmation of *osk* transported in large aggregates or oligomers, as has been theorized by others [15]. We observe these oligomers to have a defined size limit through the ring canal, and they are persistently maintained throughout the transport process (Movie S5).

### Nuclear protein Hrp48 acts to aggregate *osk*

In order to understand the processes that lead to formation of large aggregates of *oskar* mRNP we explored the role of Hrp48, a protein expressed in the nurse cell nucleus [51]. Hrp48 is essential for the correct localization of *osk* to the posterior of the oocyte, with point mutations in the N-terminal RNA-recognition motifs (RRM) and glycine-rich domains abrogating this localization [10]. An interesting consequence of some Hrp48 mutations is that Staufen protein fails to localize to the posterior of the oocyte [10]. Hrp48 along with another nuclear protein, Bruno, have been shown to bind to regions of *osk* 3′-UTR termed the Bruno response elements (BREs) [10], [14]. It has been recently proposed that Bruno's binding to the 5′- and 3′- UTRs enables the oligomerization of *osk* [15]. Since Hrp48 is a member of the hnRNP A/B family of RNA binding proteins, and binds to the same three BREs in the *osk* 3′- UTR, as well as to a translational activation element near the 5′ end of the mRNA, we examined its role in the *in vivo* oligomerization of *osk*.

**Figure 5:**
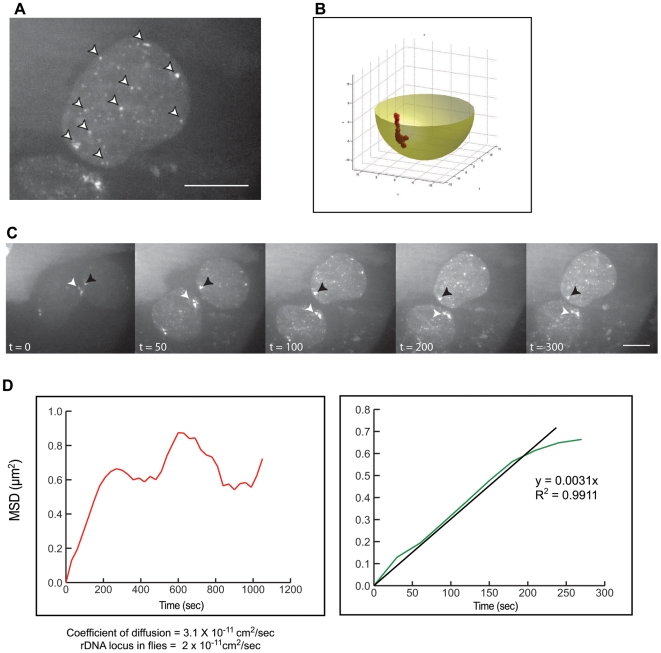
Nuclear protein Hrp48 acts to aggregate *oskar* mRNPs. (A) *oskar* mRNP nuclear dynamics in Hrp48 mutant oocytes, where distinct particles are only observed in the nucleus (arrowheads) and not in the cytoplasm of a nurse cell. (B) The particles were tracked in real-time and shown to localize to a confined location within the nuclear volume. (C) Oligomer formation of *oskar* mRNPs appear to behave like a genetic locus, i.e. ribosomal DNA genomic locus, where the MSD calculations coincide with published data. (D) The left graph is re-plotted on the right for the first 300 time points, where calculations of the diffusion coefficients are most robust. See Movie S9. Scale bars represent 10 µm. N>30.

We sought to shed light on the role Hrp48 could play in the reshaping/oligomerization process and whether the formation of oligomers was an essential precursor to localization. To this end, we imaged Hrp48 mutant egg chambers in a Staufen-GFP background (Figure 5). We observed a weak and highly diffuse pattern of *osk* in the nurse cell and oocyte cytoplasm, with no accumulation whatsoever of *osk* at the posterior pole (data not shown). Interestingly, in the nurse cell nuclei, we saw the accumulation of *osk* particles that appeared to be relatively immobile (Movie S9) as compared to wild-type (Movie S1). Further we observed that the small amounts of *osk* that were exported to the cytoplasm were unable to form particles in the cytoplasm that could then be transported to the posterior as had been observed in the wild-type background (Figure 5). The lack of *osk* particles suggested that *osk* required Hrp48 for its assembly into a large mRNP particle of oligomers and for its subsequent association with Staufen. Further, it strongly implicated the oligomerization step as a key step in its efficient transport and localization. It also supposes that binding to the BRE region of *osk* by Bruno or functional Hrp48 is essential for the aggregates of *osk* to form. Because a similar phenotype was observed in Hrp48 mutants where Staufen particles are formed (data not shown), we ruled out a direct relationship between *osk* particle formation and Staufen particle formation in Hrp48 mutants. Hrp48 is one of the three most abundant hnRNPs in *Drosophila* and is thought to bind most, if not all, transcripts in the nucleus [52], [53], [54], [55]. Therefore its effect on Staufen particle formation may be pleiotropic, and due to an effect Hrp48 has on the metabolism of other mRNA transcripts.

We further quantitatively analyzed the *in vivo* dynamics of the *osk* particles in the nurse cell nuclei of these Hrp48 mutants. We used QUIA to analyze and track single *osk* particles and reconstructed their motion in 3D (Figure 5B, Movie S9). Due to the natural motion of the nurse cell nucleus during oogenesis, we used a separate algorithm that calculates the motion of nucleus in space, and subtracts this from the motion of detected particles [56]. Using this method, we were able to quantify the precise degree of motion of *osk* and determine its degree of confinement to a specific region of the nucleus. We calculated the average MSD of the nuclear *osk* and its coefficient of diffusion (Figure 5D). Our results for *osk* particles were close to those calculated for the *Drosophila* ribosomal DNA locus, a locus confined in its mobility [57]. Ordinarily this would be highly suggestive of an mRNA that essentially behaves like a restricted transcription locus with highly constricted mobility. The oscillating motion of the particle is suggestive of a confinement to a site of transcription and an inability of *osk* to venture further away. Alternatively, the particles indicate where transcription is occurring by an accumulation of mRNA (Figure 5C). Larger particles do not form in the cytoplasm as we observe in wild-type oocytes. The latter explanation is strengthened by observations of diffuse *osk* signals in the cytoplasm, indicating that *osk* is indeed exported to the cytoplasm, but does not form particles there. Therefore Bruno and Hrp48 are necessary for *osk* oligomerization, with functional Bruno alone being insufficient.

## Discussion

To date, the subtle changes that occur at distinct steps in the transport of RNA-protein complexes, particularly *osk* and its known *trans*-acting proteins, have been impervious to biochemical analyses. The determination of the posterior pole plasm, germline and abdominal determinants, depends in large part on the Staufen-dependent localization of *oskar* mRNA. *osk*'s transport over the relatively long distance from the nurse cell to the oocyte posterior, necessitates a highly choreographed interaction with *trans*-acting factors acquired within the nucleus and the cytoplasm. By imaging *oskar* mRNA and Staufen *in vivo*, we have been able to provide biophysical evidence for several important events. Firstly, and contrary to all our expectations, *oskar* mRNA is only able to associate with Staufen near the mid-posterior of the oocyte where the two form large particles together and become co-localized to the posterior extremity in a tight crescent. Secondly, this transport occurs in higher order RNP assemblies, or aggregates, for which Hrp48 is necessary, but not sufficient, for their formation. These aggregates undergo extensive reshaping during their transport, acquiring and losing mRNA particles as they travel from the nurse cell to the posterior of the oocyte. Thirdly, as has also been recently shown by Zimyanin et al., the transport of *osk* is rapid, with the motion best described as super diffusive while in the oocyte. Our studies have aided in further refining the model for this transport posited by Zimyanin et al., which they describe as a “biased random walk”. Because we have used a bespoke 3D tracking algorithm that is able to describe local motion in different parts of the oocyte, we are able to provide an unprecedented description of the motion of several subpopulations of *osk* (Figure 3B). In general these velocities are in concordance with the most predominant findings reported in Zimyanin et al. while taking into account the smaller standard deviations. By investigating the role of microtubules and kinesin, they show that *osk* is not necessarily conjugated to kinesin for its motion in the oocyte. Thus kinesin could influence at least one phase of *osk* transport either directly, by being bound to *osk*, or indirectly by creating cytoplasmic flows. This could also explain the plethora of *trans*-acting factors *osk* interacts with, in order to regulate progression through these various steps.

### Towards a new role for Staufen

Staufen's implication in the transport of mRNAs extends to several biological systems [18]. In many aspects, the localization of *oskar* mRNA to the posterior of the oocyte can be related to the synaptic-activity dependent localization of CaMKII mRNA to the dendrites of active synapses [58]. In *Drosophila*, almost all the molecular players found in the oocyte are recapitulated in mRNA transport in *Drosophila* neurons, including Staufen [19], [58]. The mammalian homologues of Staufen, Staufen 1 and Staufen 2, have highly conserved roles in mRNA transport in neuronal cells, underlying the broad significance of understanding the interplay between Staufen and mRNA [18]. Because Staufen has been found to be important in the correct localization of *osk*, and due to their similar localization pattern in the developing oocyte, it has been used as a surrogate to indicate the transcript's location [46], [59], [60]. In those studies, *osk* transport was assumed to be dependent on Staufen, from its earliest contact until its eventual localization at the posterior of the oocyte. In contrast, we observe Staufen to be in particles distinct from *osk* until only the latest steps in the transport process near the posterior of the oocyte. This strongly suggests that Staufen protein is more likely to be implicated in the later stages of *osk* localization. Our data show that despite frequent and fleeting collisions between *osk* and Staufen at the ring canal, these still do not result in the formation of an observable stable *osk*-Staufen complex. Moreover, though *in situ* hybridization studies have shown *osk* localized to the anterior of the oocyte in Staufen mutants, we and others have observed that in Staufen mutants, *osk* is able to reach the posterior of the oocyte, but does not become highly concentrated there [20], [44].

The Macdonald group first posited [14] that the role of Staufen may not necessarily be in transport, but rather in translational activation of *osk* at the posterior. Gunkel et al., 1998 and Rongo et al., 1995 found that localization dependent translation of *osk* occurred in a highly spatially restricted manner only at the posterior and required a locally restricted interaction between *trans*-acting factors and *osk*. Indeed as Kim-Ha et al., 1995 demonstrated, of all the so-called ‘posterior group genes,’ only Staufen was able to activate translation of *osk*. Several *trans*-acting factors have been found that interact with *oskar* mRNA, and they almost universally play a role in translational repression to maintain the *osk* particle translationally silent during its long journey from the nurse cell nucleus to the posterior of the oocyte. Interestingly, a core component of the RISC (RNA induced silencing complex), *aubergine*, is required for *osk* translation, and the microRNA mir280 has been computationally predicted to bind *oskar* mRNA [61], [62], [63]. Since Staufen protein is required for translation and only interacts with *osk* near the posterior, we propose that Staufen is the *trans*-acting factor that is able to stably associate with *osk* and spatially restricts the translation of Oskar protein to the posterior of the oocyte.

Studies in neurons have also questioned the role Staufen plays in the direct trafficking of mRNPs [64]. The binding domain of *Drosophila* Staufen dsRBD3, which is essential for Staufen function and *osk* localization, shares common structural features with the N-terminal domain of bacterial ribosomal RNA-binding protein S5 [65]. This suggests that Staufen may not bind chiefly to *osk*, but rather to another component of the mRNP, as it is speculated to do in neurons. Bolstering this observation is the finding that Staufen 1 and 2 in mammals are recruited to the polysomes and not to newly synthesized cytoplasmic mRNA [64]. Taken together, the observed results suggest that Staufen-containing mRNPs could be linked to mRNA engaged in polysomes, and thus, not directly related to that of mRNA recently exported from the nucleus or in transport to those polysomes. Staufen has been suggested to play a role in actually targeting *osk* to polysomes [59]. If Staufen targeted *osk* to polysomes for translation this would also explain the observed phenotype of increased Staufen accumulation at the posterior due to increased *osk* expression [23].

### Long range RNA transport is linked to translational control

Our data in Staufen mutants begs the question of how *osk* is transported. Our findings are reinforced by the recent work of Zimyanin et al. describing the motion of *osk* to be “biased random walk”. Our biophysical data, and especially our highly refined spatial data describing specific velocities in specific regions is the first of its kind for *osk.* It largely concurs with the velocities given by Zimyanin et al., 0.04 µm/s±0.02 versus our largest class of *osk* particles, which is 0.022 µm/s. We find this transport to be super-diffusive or active transport in the oocyte, while in other compartments it occur at lesser speeds. Experiments we conducted in Hrp48 mutant oocytes strongly suggest that the nuclear and cytoplasmic aggregation of *osk* and its assembly into higher order RNPs, is an essential step in its transport during stage 7 to 10. It has been observed that the action of molecular motors, kinesin I and dynein, generate cytoplasmic flows which serve to traffic large particles such as *osk* [48], [66]. Further microtubule studies have shown a general motion of *osk* to the plus ends of the microtubule in the oocyte. Once *osk* reaches the posterior pole, it is ‘captured’ and the anchoring phase is initiated. Oskar protein acts to trap *osk* mRNA at the posterior, spatially controlling its translation, and creates a key feedback loop in the accumulation of *osk*. This may occur due to the prepositioning of ribosomes at the posterior, thus making synthesis of Oskar protein possible only there. This is true in neurons where ribosomes positioned at the post-synaptic, density to facilitate spatiotemporal control of translation of localized mRNAs.

Long-range transport of mRNA depends on nuclear factors overwhelmingly acting to repress translation during transport and spatial control of translation of these mRNAs. This may be coupled to three different levels of *osk* translational and localization control that have been hypothesized in *Drosophila* [14]. All are well in line with our current observations of Staufen-*osk* interactions, and those described by Zimyanin et al. The first would occur prior to, and independent of, posterior localization, and is characterized by the *trans*-acting factors, loaded in the nucleus and in the cytoplasm, which repress *osk* translation. The release from translational repression would be the second level. It would also require posterior localization of *osk* and as Gunkel et al., 1998, determined, a locally restricted interaction with a *trans*-acting factor would occur at the posterior of the oocyte to permit translation. Finally, the third level of translational control would occur as a translational activation event independent of both the initial repression and localization. This latter level of control is suggested by the phenotype of Staufen mutants. Under normal circumstances, posterior localization is a pre-requisite for *osk* translation. However, Staufen mutants still lack *osk* activity when the first level of control is abolished, i.e. translational repression mediated by nuclear factors such as Bruno. This implicates a requirement for Staufen in translation, independent of localization. Prior to our data, it was difficult to reconcile the ability of Staufen to be a key translational activator and be the major protein involved in the transport of *osk*. Some models of transport had suggested Oskar protein is actively degraded as *osk* is transported until it reaches the posterior where the degradation mechanism is silenced [59]. Our data, showing a localized interaction between *osk* and Staufen near the posterior, is able to reconcile the role of Staufen with its demonstrated role as a translational activator.

Staufen-*osk* interactions from our data are only stable at the mid-posterior, which still suggests an as of yet unidentified mechanism by which their stable interaction is spatially restricted to this region. A possible mechanism is the microRNA dependent recruitment of RISC to *osk*. Cook et al., 2004 suggested that mir280 binds to *osk* would recruit the RISC components *armitage (armi)* and *aubergine (aub)*. Like Staufen, these proteins are required for the timely translation of *osk*. Evidence that may link Staufen to RISC comes from the recent finding showing a high degree of conservation between the RBD of Staufen and the *Drosophila* C virus (DCV) protein [67]. DCV protein is a suppressor of RNAi and sequesters siRNA produced in an antiviral response by *Drosophila* to destroy viral RNA, thus enabling DCV to more successfully infect *Drosophila*. This high degree of conservation in the RBDs suggests Staufen may act through RISC to control *osk* translation. In addition, *armi*, an SDE3 homolog, contains eight motifs characteristic of the Upf1p family of ATP-dependent RNA helicases. In mammals, Staufen 1 recruits Upf1 to specific 3'UTRs where it acts in mRNA decay [68]. Similarly, Staufen may associate with Armi and promote *oskar* mRNA decay at the posterior pole during later stages of oogenesis.

### Higher order mRNP assemblies in the transport of mRNA

We determine that the transport process from the nurse cell nucleus to the oocyte posterior requires *osk* to be in a higher order RNP assembly that, if disrupted, leads to an inability to localize at the posterior or associate with Staufen there. Our results reveal the dynamic nature of *osk* particle assembly and movement through the various compartments of the oocyte and illuminate the dynamics of what was, until now, an opaque process. Our highly specific detection technique for mRNAs enabled for the first time, the continuous real-time, simultaneous covisualization of a native mRNA and a protein. Engaging rapid acquisition microscopy, we observed the dynamic behavior of *osk* and Staufen from a nurse cell through the ring canal and into the oocyte. We observed the formation of large mRNP particles undergoing extensive reshaping prior to and upon exiting the ring canal, all in a Staufen independent manner.

The formation of such oligomers has been recently proposed [15] as a revised version of the Spirin hypothesis [69]. Namely, that mRNAs, such as *osk*, are sequestered in large complexes ‘masking’ them from translation and maintaining their stability. Chekulaeva and coworkers have proposed that Bruno binds to recognition sites in the 5′- and 3′- UTRs of *osk*, acts to promote the oligomerization of *osk* and represses translation. Our results are the first visual confirmation of *osk* transported in such oligomers of many *osk* transcripts. Through an elegant set of experiments, Spirin proposed that in unfertilized eggs, all stored (maternal) mRNAs exist in a large ‘informosome,’ defined as the ‘masked’ form of mRNA, found in equilibrium with inactive oligoribosomes, or mRNAs loaded with ribosomes. We found these ‘informosomes’ to be absent in Hrp48 mutants, and believe that this would also hold true in Bruno mutant flies. Thus, we show *in vivo* confirmation that high order assemblies of mRNPs, through nuclear factors, play a key role in *osk* transport. Such aggregates may possess hundreds of transcripts (Supporting Information), which remain efficiently ‘masked’ for transport over long distances. Given that Bruno and Hrp48 are thought to form homodimers that permit such oligomerization [10], [15], physical constraints must exist to limit the number of transcripts in such oligomers. The ring canal may thus act as one such limitation causing the disruption of large particles, as we have observed.

Overall, the oligomerization of mRNPs is essential for *osk* to be transported by cytoplasmic motors and, flows as implicated in studies by Zimyanin et al. Such large RNA oligomers/granules serving in transport have been strongly speculated to exist in several biological systems. The study of the biophysical processes that *oskar* oligomers and Staufen undergo, are a crucial beginning in understanding how *osk* RNA and subsequently Oskar protein are spatially positioned within the developing oocyte. Combining powerful detection methods, fluorescent proteins and image analysis algorithms, it will be possible to offer novel approaches to deciphering interactions between mRNAs, their *trans*-acting factors, cytoskeletal structures and molecular motors *in vivo*.

## Materials and Methods

### *Drosophila* Stocks and Genetics

We used the following fly stocks: OreR, stau^D3^/TM3 [70] w;stau^D3^cnsP{GFP-stau7N}/Cyo [46], Hrp48^linha^ [10], Hrp48^K02647^ Hrp48^K02814,^ Hrp48^K16203^ [71]. All germline clones were generated by the FRT/FLP recombinase system [72], using the ovoD system where only homozygous mutant cells develop further than stage 4/5 of oogenesis [73]. We created a GFP-Stau,hsFLP;FRT40A-ovoD/Cyo line, crossed males to FRT-Hrp48/Cyo females and generated germline clones via heat-shocking the third instar larvae of this cross for 2 hrs at 37°C for three consecutive days.

### Molecular Beacons

Molecular beacons were designed by D. Bratu and synthesized by M. Mhlanga & C. Gouyette at the Plateforme de Synthèse d'Oligonucléotides Longs à Haut Debit (Institut Pasteur) as described in [27], [74]. Cocktail mixes of tetramethyl rhodamine(TMR)- or Cy5- labeled probes (osk76, osk352, osk964 and osk2209) were injected at 100 ng/µl concentrations into *Drosophila* oocytes or into most posterior nurse cells, coming from females of various genetic backgrounds.

### Microinjection and Confocal Microscopy

The oocytes were teased in Halocarbon oil 700 (Fisher) on a glass cover slip. Microinjections were performed using an Eppendorf manipulator and Femtojet microinjector, delivering femtoliters of the molecular beacon solution. Live microscopy was performed with a Perkin-Elmer UltraView RS Nipkow-disk confocal system controlled with PE-viewer software (version 1.0.0.9) on a Zeiss Axiovert 200 inverted microscope with 40× objective (Zeiss PlanNeofluar, 1.3 NA, oil immersion) acquired by a Hamamatsu Orca II ER cooled CCD camera or an Andor EMCCD camera. Chromatic aberration was verified before image capture by alignment of 15 µm Focal Check Fluorescent Microspheres (Molecular Probes). With this objective, pixel size is 164.83 nm. All analysis was done in 3D with a minimum of 12 Z-stacks and 1 µm Z-step used. Exposure time was 200 ms at excitation wavelength 488 nm and 568 nm/647 nm taken in continuous acquisition mode (i.e. no pause between Z stacks). Acquired images were converted from RAW format to 16-bit TIFF using a custom made ImageJ javascript written by M. Marchand, E.Glory & M. Mhlanga. Image analysis on these TIFF files such as 3D detection and tracking of *osk* and Staufen-GFP were performed using QUIA software (Institut Pasteur) as described in the Supplementary Data. All microinjection and microscopy experiments were done at room temperature.

## Supporting Information

Data S1 Supplemental data information
(0.24 MB DOC)

Figure S1 Distribution of oskar molecular beacon in a stage 9 wild type oocyte. Single optical section of oocyte chamber, microinjected at the anterior with an oskar-specific molecular beacon labeled with TMR. Upon microinjection, the probe diffuses rapidly throughout the cytoplasm. Images were acquired every 10 sec. for 30 min. Within 100 sec., the fluorescent signal appears brightly at the posterior pole, indicating the presence of already localized oskar transcript. We quantified the fluorescent level emitted by the molecular beacon over a region encompassing the entire length of the oocyte (boxed region in first panel; graph, b). The anterior signal decreases upon diffusion of the probe from the injection site, and increases almost spontaneously to maximum levels, reflecting a thorough detection of the osk population at the posterior. N = 5. Posterior is oriented to the right. Scale bar is 25 µm.
(2.82 MB EPS)

Figure S2 Particle reshaping. Oskar mRNP imaged at the ring canal as it transverses into the oocyte. A Z-projection of 1 µm optical slices is displayed as a time sequence. The montage shows the accumulation of particles into a large RNP aggregate, which changes in shape as it funnels through the ring canal. The aggregate breaks down and dissipates as it progresses into the oocyte cytoplasm. See Video 8. Scale bar is 10 µm.
(30.32 MB EPS)

Figure S3 Barentsz and oskar. To visualize clearly the association between osk and Barentsz the red and green channels were de-noised. Cancellations of the background and noise effects make the superposition of the two channels particularly demonstrative of the colocalization at a given time. This process involves first a multi scales B3 wavelet transform which is especially adapted to isotropic signals such as RNA particles, and then a statistical thresholding in each band. Here six tracks of oskar-Barentsz-GFP complexes are shown with their movements in the cytoplasm of the nurse cell. Barentsz is present at the nuclear envelope (most likely the outer envelope) and loads oskar mRNPs as they exit the nucleus (see movie 12). The Barentsz GFP transgenic fly also expresses endogenous (unlabeled) Barentsz protein, therefore particles of oskar mRNP without Barentsz GFP are visible.
(1.48 MB DOC)

Figure S4 3D plus time tracking of oskar mRNA. (A) The velocity and MSD of osk were calculated in a wild-type oocyte injected with molecular beacons. The resulting tracks were superimposed on the final timepoint image. Using an advanced version of our standard algorithm QUIA, three regions of the oocyte were selected to analyze the velocity of osk. Osk motion is heterogenous with many molecules engaged in slow motion as well as a small sub-population engaged in rapid motion at a given time. Three regions within the oocyte are arbitrarily selected, and depicted in Green, Red and Yellow boxes. (B) The tracks within each of the Green, Red and Yellow regions were analyzed for their velocity. The number of displacements refers to the number of tracks. The tracking algorithm is able to give important information as to how subpopulations of osk particles behave in differing regions of the oocyte. This is especially important since the movement is not uniform throughout the entire oocyte chamber. Posterior is oriented to the right. Scale bar is 25 µm.
(1.00 MB PDF)

Movie S1 Nuclear export of oskar mRNPs from nucleus of a nurse cell at stage 8 of oogenesis. Molecular beacons were injected in a nurse cell cytoplasm. As the probes diffused into a neighboring nurse cell via a ring canal, they became sequestered into its nucleus. Native oskar mRNPs were visualized as they formed and exited that nucleus into the cytoplasm. Red arrowheads at time frame 440 sec mark some of the particles. Still frames with tracks are portrayed in Figure 1.
(0.96 MB MOV)

Movie S2 Oskar mRNP traffic in stage 9 oocytes. Oskar mRNP particles form in the nurse cell and are transported into oocyte via the ring canals. Arrowheads indicate several particles during an early time-point frame (60 sec). Fluorescent signal accumulates in a crest at the posterior of the oocyte indicating the localization of endogenous oskar mRNP indicated by another arrowhead that appears during a few late time-point frames of the movie (1400 sec). Time is noted on the top right corner. More details are found in legend of Figure 1.
(0.93 MB MOV)

Movie S3 Multidimensional tracking of oskar mRNPs of the sequence in Movie 1 using QUIA (supplemental data). The oskar mRNPs are indicated by different color balls with the respective tracks. This representation of the 4D reconstruction enables visualization of several particles moving between the nucleus and the cytoplasm.
(1.11 MB MOV)

Movie S4 In vivo dynamics of oskar mRNPs over the entire egg chamber. Several red arrowheads indicate the formation of oskar mRNP particles in the nurse cells and as they are transported into the oocyte. White arrows close to the dorsal and ventral cortexes and towards the posterior indicate the direction of mRNP movement within the oocyte. A red arrow throughout the movie marks the posterior location where oskar mRNP arrests in transport prior to reaching the posterior as has been observed in Bratu et al., 2003.
(1.06 MB MOV)

Movie S5 Covisualization of oskar mRNP and Staufen-GFP. Z-projections of 12×1.2 µm optical slices played over 90 min are represented in a red (oskar mRNP), a green (Staufen-GFP) and merged-colors time-lapse movies. The merged representation highlights the temporal colocalization of oskar mRNP with Staufen-GFP throughout the egg chamber. A zoomed view of the merged movie enables the colocalization Staufen and oskar mRNP to be observed only near the posterior of the oocyte, contrary to expectations. The dynamic movement of red and green particles persists throughout the egg chamber during the time of the acquisition.
(3.13 MB MOV)

Movie S6 In vivo dynamics of oskar mRNP is Staufen mutant oocytes. The oskar mRNP is able to reach all parts of the oocyte. QUIA generates tracking data for each detected particle that meets minimum displacement and speed criteria predetermined by the user. Yellow tracks are shown only for those detected oskar mRNPs that are displaced at least 2.5 µm in distance. Figure 4.
(1.40 MB MOV)

Movie S7 Oskar mRNP transport through the ring canal. The passage of oskar mRNP as it remodels to cross the ring canal from the nurse cell into the oocyte. Many particles are detected across the ring canal and 4 are numbered to correspond to the analysis data in Figure 5.
(1.97 MB MOV)

Movie S8 oskar mRNP and Staufen behavior at the ring canal. Z-projections of 12×1.2 µm optical slices played over 30 min; Staufen (green) and oskar mRNP (red) are shown to undergo numerous collision that do not result in stable associations once they exit the ring canal and enter the oocyte. Extensive reshaping of oskar mRNP is also observed as a big particle funnels through the canal and breaks down into smaller particles within the oocytes.
(1.32 MB MOV)

Movie S9 Oskar mRNP dynamics in Hrp48 mutant oocytes. Oskar mRNPs are seen as small particles in the nurse cell nucleus. The particles undergo movements within the nucleus but are not seen exiting as in Movie 1. Arrowheads indicate oskar mRNPs. Colored tracks of individual particles are shown in parallel; the circle represents the last time point on the track.
(2.24 MB MOV)

Movie S10 Models of colocalization estimation. Movie 10 has a model of a synthetic sequence with two colored “blobs” that cross each other several times. Movie 11 is the identical sequence with noise and background signals added to the simulation. This is further described in the Supporting information section.
(0.15 MB MOV)

Movie S11 Models of colocalization estimation. Movie 10 has a model of a synthetic sequence with two colored “blobs” that cross each other several times. Movie 11 is the identical sequence with noise and background signals added to the simulation. This is further described in the Supporting information section.
(3.78 MB MOV)

Movie S12 4D representation of Barentsz-GFP and oskar mRNA in stage 8 egg chamber. Barentsz protein (green) localizes at the nuclear envelope in the nurse cells. It is seen loading on oskar mRNA (red) and maintaining a stable association with oskar mRNA in time.
(2.37 MB MOV)
