# Supplementary material for: In Vivo Colocalisation of oskar mRNA and Trans-Acting Proteins Revealed by Quantitative Imaging of the Drosophila Oocyte
Source: PLoS One. 2009 Jul 14;4(7):e6241. doi: 10.1371/journal.pone.0006241 (PMC2705681; doi:10.1371/journal.pone.0006241)
Supplement: Data S1 — Supplemental data information (0.24 MB DOC) [file pone.0006241.s001.doc]

# Supporting Information

**Analysis of Tracking Data in *Drosophila* oocytes**

Brownian motion analysis is applicable to diffusive movements such as those of RNA, DNA and proteins. To access the laws underlying the motion of mRNA they can be analyzed by computing the mean squared displacement (MSD) in 3D of a tracked particle as a function of a time interval, ∆d in µm and ∆t in seconds:

<∆d^2^> = <(X(t)-X(t+∆t))^2^>+<(Y(t)-Y(t+∆t))^2^>+<( Z (t)- Z (t+∆t))^2^>

The average is performed over all overlapping time intervals. In our analysis, the X, Y and Z coordinates of the tracked mRNA are obtained via QUIA (the detection algorithm). In instances when the cell nucleus is moving over time (Figure 5), we subtract the position vector of the nucleus centroid from the position vector of the tracked mRNA particles to obtain the movement of tracked *osk* mRNA relative to the nucleus.

Previous studies have described the dynamics of mRNA in the nucleus to be consistent with free diffusion (Gorski et al., 2006; Shav-Tal et al., 2004; Vargas et al., 2005). Our data is similar, finding the nuclear component of *osk* mRNA motion to be free-diffusion (Figure 1D). But unlike the nucleus, the nurse cell and oocyte chamber were poorly described by normal diffusion. The MSD of these compartments (oocyte & nurse cell) are better fitted to a power law. A power law is more visibly identifiable on a log-log plot. To highlight these differences, we represented the MSD curves on both linear and log plots (Figure 1D). The linear character of the curves on a log-log scale indicates a power law dependence of MSD with time: <∆d^2^> = C ∆t^α^. We compute C and α by fitting this power law to the empirical MSD curves as shown in the log-log plot in Figure 1D. The exponents α extracted from power law fitting the MSD then reflect the motion dynamics: α = 1 is consistent with Brownian motion, α > 1 reflects super-diffusion and α < 1 is consistent with sub-diffusion. To ensure the robustness of the computed parameters C and α, we performed the fits for time intervals up to 30% of the total duration of the observation for at least 40 tracks.

We observed, as have other groups (Glotzer et al., 1997; Palacios and St Johnston, 2002; Serbus et al., 2005), that the underlying motion in the oocyte chamber is of a cytoplasmic flow or stream. This implies the *osk* motion we measure is either superimposed or dependent on such flows. Future studies of *osk* dynamics will need to take into consideration (i.e. via modeling) the fluid motion/stream in the oocyte to ascertain its contribution to the motion we have described here.

**Diffusion and Hybridization Kinetics of Molecular Beacons within the Oocyte**

To determine the rate of diffusion of molecular beacon probes within the *Drosophila* oocyte, we microinjected a solution of a TMR-labeled molecular beacon, designed to bind to the coding region of *osk*, to the anterior of a stage 9 oocyte. At this developmental stage, the oocyte’s size is approximately 50% of the entire egg chamber, with the greater population of *osk* transcript already localized at the posterior pole. Within seconds after microinjection, images of a central optical slice were acquired every 10 sec for 20 min (Figure S1). We quantified the distribution of the fluorescent signal across the length of the oocyte, finding that the probe diffuses from the injection site to the cortex very quickly (~30 sec post injection). The posterior signal appears within 1 minute and, while becoming brighter over time, it reaches an intensity 100% more than that found at the anterior (Figure S1B). The maximum fluorescent signal is evident after just 10 minutes of imaging, indicating that a limiting factor (probe or target) is involved in the reaction.

**Particle remodeling**

Images acquired across the ring canal provided insight in the dynamic makeup of the *osk* aggregates as they are transported between the two chambers. Optical slices projected over time (Figure S2) captured a process never before seen of *osk* mRNP particles accumulating at the ring canal, funneling through while adjusting in size, and dispersing into smaller fractions once in the oocyte’s cytoplasm. This volumetric check point may very well be distinctly connected with the onset of cytoplasmic flows in the oocyte when the nurse cell material becomes dumped into the oocyte during mid oogenesis.

**Molecular Beacons vs. Fluorescently Labeled *oskar* RNA**

Previous *in vivo* studies of *osk* localization involved injecting *in vitro* transcribed fluorescently labeled *osk* and imaging its distribution throughout the egg chamber (Glotzer et al., 1997). Unfortunately, this approach did not provide many findings about the transport and localization mechanisms in the oocyte, except *osk*’s need to undergo nuclear processing for proper localization to occur at the posterior pole (Hachet and Ephrussi, 2004). To confirm that molecular beacons hybridize to endogenous *osk*, we co-injected *in vitro* transcribed ROX-labeled *oskar* RNA with an *oskar*-specific molecular beacon labeled with Cy-5 into a wild type nurse cell, and observed their behavior every 10 sec over 20 min. Upon injection, a population of probes hybridized to the transcribed RNA, forming particles almost instantaneously (Figure 1). Unbound probes were free to diffuse and become sequestered within the nucleus in minutes (Figure 1, green in Merge). In the oocyte, they bound tightly to localized endogenous *osk* at the posterior pole (Figure 1, Cy-5 inset). The *oskar*-ROX signal was distributed throughout the nurse cell cytoplasm, never in the nucleus, and with very little accumulation in the oocyte. As *in vitro* transcribed *osk* evades nuclear processing, it failed to localize at the posterior pole (Figure 1, ROX inset) with the endogenously expressed *osk*. Thus molecular beacons offer a unique approach for detecting and studying the processes that *oskar* mRNA undergoes from the moment it exits the nurse cell nucleus until it localizes tightly to the posterior pole in the oocyte.

**Multiple Particle Tracking in 4 Dimensions (xyz+t)**

For the purpose of measuring the dynamic properties of RNA particles in 3D space, we developed our own image analysis algorithm. This was necessary as moving particles change their behavior and shape over time. The method combined a wavelet-based detection (Olivo-Marin, 2002) an Interacting Multiple Model estimator with adapted models (Genovesio et al., 2006), a Split and Merge data association and a self-adaptive gating mechanism (Genovesio et al., 2004). All four were developed at the Pasteur Institute and implemented in a dedicated software program called QUIA. The method has the advantage of being able to track multiple objects in complex situations where they cross paths or temporarily split. We first developed a model using synthesized data to confirm that this algorithm enables robust tracking of a density of targets. In brief, the method enables the extraction and analysis of information such as the number, position, speed, movement and diffusion phases of individual particles. A detection stage is performed by applying a three-dimensional, undecimated wavelet transform. Consequently we compute, for each detected signal, several predictions of its state within the next frame. This is accomplished via an Interacting Multiple Model (IMM) algorithm, which includes several models corresponding to different biologically realistic types of movement. The tracks are then constructed by a data association algorithm based on the maximization of the likelihood of each IMM. The final stage involves updating the IMM filters in order to compute final estimations for an existing image and to optimize predictions for the next image.

**Colocalization Estimation Method**

We sought to establish a measurement of the amount of fluorophore at certain locations simultaneously in two wavelengths: one for Staufen GFP-(A) and the other for *osk* -(B) labeled by molecular beacons. A voxel (x,y,z) will have a high colocalization factor when both wavelength A and wavelength B have high levels of intensity, or a low colocalization factor when either or both A and B have low levels of intensity. The two wavelengths are then multiplied. Furthermore, to limit the effect of the difference of scale between two bands, each 3D image at each time step t and for each band is centered and normalized. The mean over all voxels of a certain sub-volume gives the colocalization value in that sub-volume. Thus:

- The positive centered reduced value of a voxel in wavelength A is:

- The colocalization factor of a voxel is
- The colocalization factor for a sub-3D image is

To validate our method, we produced two synthetic sequences each containing a ‘blob’ that moves. The two ‘blobs’ cross one another several times across a sequence.

40
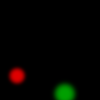

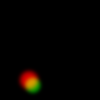
 57

The following graph represents the values of their colocalization factor with time
(Supplementary Information, Video 10).

The highest peak corresponds exactly with the event produced when one ‘blob’ perfectly overlaps another. Lower peaks correspond to image frames when only a fraction of a ‘blob’ is superimposed on another (i.e. Image #57).

When noise and background signals are added to the simulation, the colocalization factor become embedded into those signals.

40
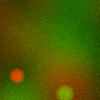

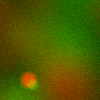
 57

This effect is demonstrated by the pink curve in the following graph (Movie 11).

To minimize the contribution of these signals when calculating colocalization factors, the background volume **M** obtained by averaging all the sequences of each image before processing the colocalization factor is subtracted. Therefore:

- The background volume for wavelength A is computed by
- The foreground image is then extracted and replaces the original image as describe by $I_{A}=I_{A}-M_{A}$
- A corrected image is likewise generated for wavelength B.

The resulting colocalization profile, represented in yellow, denotes once again the peaks where the two signals generated by the ‘blobs’ colocalize.

This method was employed for comparing the variation of colocalization factors through different parts of a volume.

# Determining the Number of *osk* Transcripts per Detected Particle

We estimate the number of *osk* transcripts to be in a range of 300 to 500 per particle we observed. The number of transcripts we theoretically calculated is ten fold higher (see following section on “Correction for *trans*-acting proteins bound to mRNA”).

Though several assumptions were made as the number of *osk* per particle was calculated, we believe that the approximated result cannot be more precisely determined given the current instruments and techniques available. In addition we make assumptions similar to those made in the literature (Shav-Tal et al., 2004; Vargas et al., 2005).

We first assume that the RNA intensity profile is Gaussian distributed and isotropic. The Gaussian standard deviation is denoted as σ. The Point Spread Function (PSF) of a Spinning Disk Confocal Microscope (SDCM) can be well approximated by a separable Gaussian function (Zhang et al., 2006), where its lateral and axial standard deviations are denoted as σ_ρ_ and σ_z_, respectively. The observed RNA intensity distribution should also be Gaussian with lateral and axial standard deviations respectively as σ_1_ = (σ^2^ + σ_ρ_^2^)^1/2^ and σ_2_ = (σ^2^ + σ_z_^2^)^1/2^.

The observed intensity is thresholded to determine the RNA particle’s basal fluorescent intensity. Due to the Gaussian intensity profile, the resulting volume (*V_s_*) is ellipsoid and it is assumed to be collected via a 95%-total-intensity clipping:

(1)

The true RNA volume is then calculated as:

(2)

(2)

The expressions of σ_ρ_ and σ_z_ for SDCM equipped with small pinholes are given by:

(3)

(4)

where:

- λ*_ex_* and λ*_em_* are the excitation and emission wavelengths respectively in SDCM,
- *n* is the refractive index,
- α is the maximal semi-convergence angle of the objective of SDCM, which is derived from the numerical aperture (NA).

In our experiments, the acquisition parameters are given by:

λ*_ex_* = 546nm ; λ*_em_* = 568nm; *n* = 1.515 and NA = 1.3

Substituting these values into Eq. (3) and (4), we calculated the lateral and axial standard deviations to be σ_ρ_ ≈ 66.41nm and σ_z_ ≈ 209.51nm.

The value for *Vs* is determined by observations and derivations via the QUIA program using fluorescent intensity inputs. We have predetermined the fluorescent intensity generated by a single molecular beacon upon binding to an *osk* using the method introduced by Vargas et al., 2005.

Thus the value for σ is solved via equation (1) with *Vs* obtained from QUIA and the σ_ρ_ and σ_z_ values calculated for both wavelengths. Consequently, the true volume *V_o_* is calculated using equation (2). The volume of one *oskar* mRNA is predicted by using a compact spherical volume formula:

where:

- *N* is the total number of nucleotides in an *oskar* mRNA (2870),
- *r* is the radius of one nucleotide (~ 0.3nm)

Finally, the total number of *oskar* RNAs in one particle is estimated by dividing *V_O_* by *V_R_*. For an observed particle volume of 2µm^3^, we calculated the presence of 5000 transcripts. Assuming an overestimation of one log (see below “Correction for *trans*-acting proteins bound to naked *oskar* mRNA”), the total number is refined to be ~500 transcripts.

**Correction for *trans*-acting proteins bound to naked *oskar* mRNA**

The above calculation takes into consideration *oskar* mRNA devoid of any association with *trans*-acting proteins, therefore a ‘naked’ transcript. Endogenous *oskar* mRNP is initially composed of the exon junction complex (EJC) consisting of a minimum of seven proteins bound to three splice junctions. Each EJC has a mass of ~400kDa representing a total mass of ~1200kDa (Dreyfuss et al., 2002). Without taking into account other *trans*-acting proteins known to bind *oskar* mRNA, a consideration for this minimal correction for the mRNP size must be made. Due to the transient nature of the interactions by non-EJC proteins and the absence of a clearly resolved NMR structure of the EJC bound to an mRNA, the mathematical volume correction described above did not include the volume contributions from these proteins. We believe we overestimated the particle volumes by a factor of one logarithm and therefore reduced our total RNA prediction by this factor.

# Staufen-GFP flies

The Staufen GFP flies were developed in flies containing no functional endogenous Staufen (Martin et al., 2003). Thus far no experimental evidence exists clearly indicating that the Staufen-GFP in these transgenic lines has differing biological activity to that of unlabeled Staufen. Indeed the addition of GFP to Staufen has been shown to preserve the functionality of the protein retaining normal localization and leaving the posterior localization of *osk* unaltered. This is evidenced by its extensive use to perform screens for genes affecting *osk* and Staufen localization.

# Image De-noising and Particle Tracking in 3D to study co-localization of Barentsz GFP and *oskar* mRNA

To visualize clearly the association between *oskar* mRNA and Barentsz, the red and green channels were de-noised (Figure S3). Cancellations of the background and noise effects make the superposition of the two channels particularly demonstrative of the colocalization at a given time. This process involves a multi-scales B3 wavelet transform that is especially adapted to isotropic signals, such as RNA particles, and a statistical thresholding in each band (Starck et al., 1998). The noiseless image is then obtained by a reconstruction from the thresholded scales.

To study the consistency of colocalization of the observed particles, we chose to track them independently in separate channel images (Figure S3E). Particles were detected in each channel by thresh-holding the appropriate de-noised image. Based on these detections, particles were tracked by an Interacting Multiple Model estimator (Genovesio et al., 2006), which is specifically adapted to include several kinetic models that correspond to biologically realistic types of movements (i.e. Brownian motion or directed motion). The tracks were then selected by a data association algorithm, which maximizes the likelihood of each IMM.

We obtained one set of tracks for each channel, which we then compared by superposition of the two noiseless images and the simultaneous drawing of the tracks. Additional information such as the number, position and speed of individual particles are computed for each set of tracks, allowing for further automatic comparisons. For example, distances between tracks at each time step can be computed, allowing us to show that some of them stay very close for many frames, indicating that the association between corresponding particles is stable over time.

This overall denoising-detection-tracking procedure was developed at the Pasteur Institute and implemented in QUIA.

## Supplementary Figure Legends

## Supplementary Figure 1

**Distribution of *oskar* molecular beacon in a stage 9 wild-type oocyte. (A)** Single optical section of oocyte chamber, microinjected at the anterior with an *oskar*-specific molecular beacon labeled with TMR. Upon microinjection, the probe diffuses rapidly throughout the cytoplasm. Images were acquired every 10sec. for 30min. Within 100sec., the fluorescent signal appears brightly at the posterior pole, indicating the presence of already localized *oskar* transcript. **(B)** We quantified the fluorescent level emitted by the molecular beacon over a region encompassing the entire length of the oocyte (boxed region in first panel in **(A)**). The anterior signal decreases upon diffusion of the probe from the injection site, and increases almost spontaneously to maximum levels, reflecting a thorough detection of the *osk* population at the posterior. Posterior is oriented to the right. Scale bar is 25µm. N=5.

## Supplementary Figure 2

**Particle reshaping.** *Oskar* mRNP imaged at the ring canal as it transverses into the oocyte. A Z-projection of 1µm optical slices is displayed as a time sequence. The montage shows the accumulation of particles into a large RNP aggregate, which changes in shape as it funnels through the ring canal. The aggregate breaks down and dissipates as it progresses into the oocyte cytoplasm. Each panel is 3 seconds apart. See Movie 8. Scale bar is 10µm.

## Supplemental Figure 3

**Barentsz and *oskar* mRNA.** **(A)** To visualize clearly the association between *oskar* mRNA and Barentsz, the red and green channels were de-noised. Cancellations of the background and noise effects make the superposition of the two channels particularly demonstrative of the colocalization at a given time. This process involves first a multi scales B3 wavelet transform that is especially adapted to isotropic signals such as RNA particles, and then a statistical thresholding in each band. **(B)** Six tracks of *oskar*-Barentsz-GFP complex movement in the cytoplasm of the nurse cell. Barentsz is present at the nuclear envelope (most likely the outer envelope) and loads *oskar* mRNPs as they exit the nucleus (See Movie 12). The Barentsz-GFP transgenic fly also expresses endogenous (unlabeled) Barentsz protein, therefore particles of *oskar* mRNP without Barentsz-GFP are visible.

## Supplemental Figure 4

**3D plus time tracking of *oskar* mRNA.** **(A)** The velocity and MSD of *osk* were calculated in a wild-type oocyte injected with molecular beacons. The resulting tracks were superimposed on the final timepoint image. Using an advanced version of our standard algorithm QUIA, three regions of the oocyte were selected to analyze the velocity of *osk*. *Osk* motion is heterogenous with many molecules engaged in slow motion as well as a small sub-population engaged in rapid motion at a given time. Three regions within the oocyte are arbitrarily selected, and depicted in Green, Red and Yellow boxes. **(B)** The tracks within each of the Green, Red and Yellow regions were analyzed for their velocity. The number of displacements refers to the number of tracks. The tracking algorithm is able to give important information as to how subpopulations of *osk* particles behave in differing regions of the oocyte. This is especially important since the movement is not uniform throughout the entire oocyte chamber. Posterior is oriented to the right. Scale bar is 25µm.

**Movie Legends**

**Movie 1** – Nuclear export of *oskar* mRNPs from nucleus of a nurse cell at stage 8 of oogenesis. Molecular beacons were injected in a nurse cell cytoplasm. As the probes diffused into a neighboring nurse cell via a ring canal, they became sequestered into its nucleus. Native *oskar* mRNPs were visualized as they formed and exited that nucleus into the cytoplasm. Red arrowheads at time frame 440sec mark some of the particles. Still frames with tracks are portrayed in Figure 1.

**Movie 2** – *Oskar* mRNP traffic in stage 9 oocytes. *Oskar* mRNP particles form in the nurse cell and are transported into oocyte via the ring canals. Arrowheads indicate several particles during an early time-point frame (60sec). Fluorescent signal accumulates in a crest at the posterior of the oocyte indicating the localization of endogenous *oskar* mRNP indicated by another arrowhead that appears during a few late time-point frames of the movie (1400sec). Time is noted on the top right corner. More details are found in legend of Figure 1.

**Movie 3** – Multidimensional tracking of *oskar* mRNPs of the sequence in Movie 1 using QUIA (supplemental data). The *oskar* mRNPs are indicated by different color balls with the respective tracks. This representation of the 4D reconstruction enables visualization of several particles moving between the nucleus and the cytoplasm.

**Movie 4** – *In vivo* dynamics of *oskar* mRNPs over the entire egg chamber. Several red arrowheads indicate the formation of *oskar* mRNP particles in the nurse cells and as they are transported into the oocyte. White arrows close to the dorsal and ventral cortexes and towards the posterior indicate the direction of mRNP movement within the oocyte. A red arrow throughout the movie marks the posterior location where *oskar* mRNP arrests in transport prior to reaching the posterior as has been observed in Bratu et al., 2003.

**Movie 5** – Covisualization of *oskar* mRNP and Staufen-GFP. Z-projections of 12X1.2µm optical slices played over 90 min are represented in a red (*oskar* mRNP), a green (Staufen-GFP) and merged-colors time-lapse movies. The merged representation highlights the temporal colocalization of *oskar* mRNP with Staufen-GFP throughout the egg chamber. A zoomed view of the merged movie enables the colocalization Staufen and *oskar* mRNP to be observed only near the posterior of the oocyte, contrary to expectations. The dynamic movement of red and green particles persists throughout the egg chamber during the time of the acquisition.

**Movie 6** – *In vivo* dynamics of *oskar* mRNP is Staufen mutant oocytes. The *oskar* mRNP is able to reach all parts of the oocyte. QUIA generates tracking data for each detected particle that meets minimum displacement and speed criteria predetermined by the user. Yellow tracks are shown only for those detected *oskar* mRNPs that are displaced at least 2.5µm in distance. Figure 4.

**Movie 7** – *Oskar* mRNP transport through the ring canal. The passage of *oskar* mRNP as it remodels to cross the ring canal from the nurse cell into the oocyte. Many particles are detected across the ring canal and 4 are numbered to correspond to the analysis data in Figure 5.

**Movie 8** – *oskar* mRNP and Staufen behavior at the ring canal. Z-projections of 12X1.2µm optical slices played over 30 min; Staufen (green) and *oskar* mRNP (red) are shown to undergo numerous collision that do not result in stable associations once they exit the ring canal and enter the oocyte. Extensive reshaping of *oskar* mRNP is also observed as a big particle funnels through the canal and breaks down into smaller particles within the oocytes.

**Movie 9** – *Oskar* mRNP dynamics in Hrp48 mutant oocytes. *Oskar* mRNPs are seen as small particles in the nurse cell nucleus. The particles undergo movements within the nucleus but are not seen exiting as in Movie 1. Arrowheads indicate *oskar* mRNPs. Colored tracks of individual particles are shown in parallel; the circle represents the last time point on the track.

**Movie 10 & 11** – Models of colocalization estimation. Movie 10 has a model of a synthetic sequence with two colored “blobs” that cross each other several times. Movie 11 is the identical sequence with noise and background signals added to the simulation. This is further described in the Supporting Information section.

**Movie 12** – 4D representation of Barentsz-GFP and *oskar* mRNA in stage 8 egg chamber. Barentsz protein (green) localizes at the nuclear envelope in the nurse cells. It is seen loading on *oskar* mRNA (red) and maintaining a stable association with *oskar* mRNA in time.

References

Bratu, D.P., Cha, B.J., Mhlanga, M.M., Kramer, F.R., and Tyagi, S. (2003). Visualizing the distribution and transport of mRNAs in living cells. Proc Natl Acad Sci U S A *100*, 13308-13313.

Dreyfuss, G., Kim, V.N., and Kataoka, N. (2002). Messenger-RNA-binding proteins and the messages they carry. Nat Rev Mol Cell Biol *3*, 195-205.

Genovesio, A., Belhassine, Z., and Olivo-Marin, J. (2004). Adaptive gating in Gaussian Bayesian multi-target tracking. Image Processing, 2004 ICIP'04 2004 International Conference on *1*.

Genovesio, A., Liedl, T., Emiliani, V., Parak, W.J., Coppey-Moisan, M., and Olivo-Marin, J.C. (2006). Multiple particle tracking in 3-D+t microscopy: Method and application to the tracking of endocytosed quantum dots. Ieee T Image Process *15*, 1062-1070.

Glotzer, J.B., Saffrich, R., Glotzer, M., and Ephrussi, A. (1997). Cytoplasmic flows localize injected oskar RNA in Drosophila oocytes. Curr Biol *7*, 326-337.

Gorski, S.A., Dundr, M., and Misteli, T. (2006). The road much traveled: trafficking in the cell nucleus. Curr Opin Cell Biol.

Hachet, O., and Ephrussi, A. (2004). Splicing of oskar RNA in the nucleus is coupled to its cytoplasmic localization. Nature *428*, 959-963.

Martin, S.G., Leclerc, V., Smith-Litiere, K., and St Johnston, D. (2003). The identification of novel genes required for Drosophila anteroposterior axis formation in a germline clone screen using GFP-Staufen. Development *130*, 4201-4215.

Olivo-Marin, J. (2002). Extraction of spots in biological images using multiscale products. Pattern Recognition *35*, 1989-1996.

Palacios, I.M., and St Johnston, D. (2002). Kinesin light chain-independent function of the Kinesin heavy chain in cytoplasmic streaming and posterior localisation in the Drosophila oocyte. Development *129*, 5473-5485.

Serbus, L.R., Cha, B.J., Theurkauf, W.E., and Saxton, W.M. (2005). Dynein and the actin cytoskeleton control kinesin-driven cytoplasmic streaming in Drosophila oocytes. Development *132*, 3743-3752.

Shav-Tal, Y., Darzacq, X., Shenoy, S.M., Fusco, D., Janicki, S.M., Spector, D.L., and Singer, R.H. (2004). Dynamics of single mRNPs in nuclei of living cells. Science *304*, 1797-1800.

Starck, J., Murtagh, F., and Bijaoui, A. (1998). Image Processing and Data Analysis: The Multiscale Approach (Cambridge University Press).

Vargas, D.Y., Raj, A., Marras, S.A., Kramer, F.R., and Tyagi, S. (2005). Mechanism of mRNA transport in the nucleus. Proc Natl Acad Sci U S A *102*, 17008-17013.

Zhang, B., Zerubia, J., and Olivo-Marin, J. (2006). A study of Gaussian approximations of fluorescence microscopy PSF models. Proceedings of SPIE *6090*, 60900K.
