# Supplementary figures and images for: In Vivo Colocalisation of oskar mRNA and Trans-Acting Proteins Revealed by Quantitative Imaging of the Drosophila Oocyte
Source: PLoS One. 2009 Jul 14;4(7):e6241. doi: 10.1371/journal.pone.0006241 (PMC2705681; doi:10.1371/journal.pone.0006241)

A.

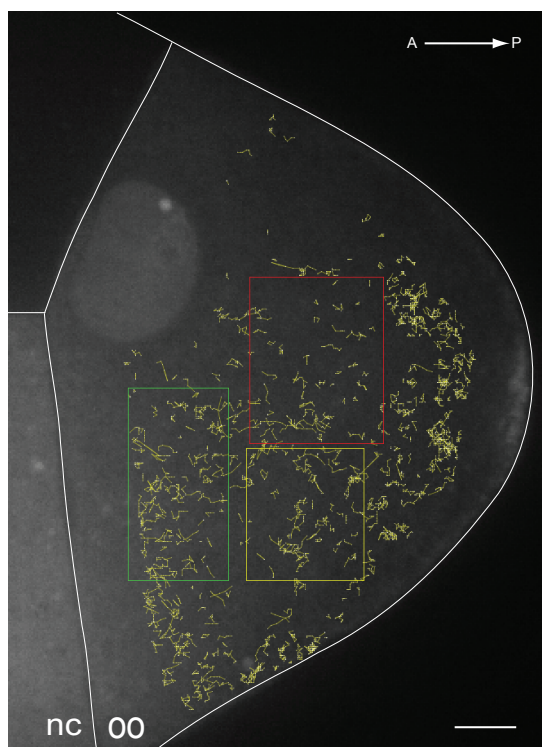

B.

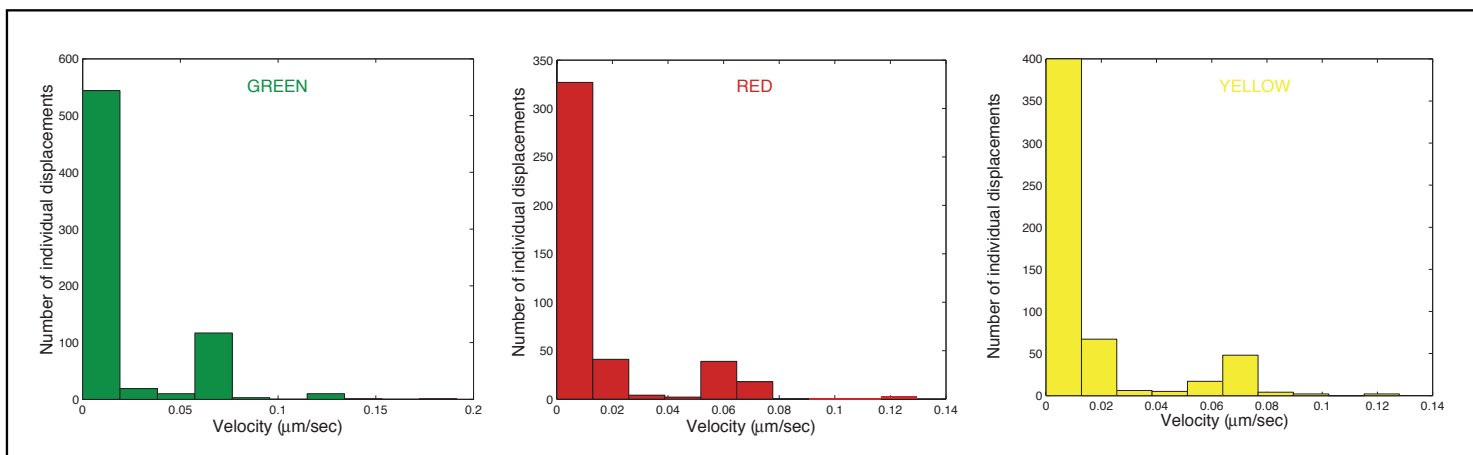

Supplement: Figure S4 — 3D plus time tracking of oskar mRNA. (A) The velocity and MSD of osk were calculated in a wild-type oocyte injected with molecular beacons. The resulting tracks were superimposed on the final timepoint image. Using an advanced version of our standard algorithm QUIA, three regions of the oocyte were selected to analyze the velocity of osk. Osk motion is heterogenous with many molecules engaged in slow motion as well as a small sub-population engaged in rapid motion at a given time. Three regions within the oocyte are arbitrarily selected, and depicted in Green, Red and Yellow boxes. (B) The tracks within each of the Green, Red and Yellow regions were analyzed for their velocity. The number of displacements refers to the number of tracks. The tracking algorithm is able to give important information as to how subpopulations of osk particles behave in differing regions of the oocyte. This is especially important since the movement is not uniform throughout the entire oocyte chamber. Posterior is oriented to the right. Scale bar is 25 µm. (1.00 MB PDF) [file pone.0006241.s005.pdf]
